# Prediction models for dementia and neuropathology in the oldest old: the Vantaa 85+ cohort study

**DOI:** 10.1186/s13195-018-0450-3

**Published:** 2019-01-22

**Authors:** Anette Hall, Timo Pekkala, Tuomo Polvikoski, Mark van Gils, Miia Kivipelto, Jyrki Lötjönen, Jussi Mattila, Mia Kero, Liisa Myllykangas, Mira Mäkelä, Minna Oinas, Anders Paetau, Hilkka Soininen, Maarit Tanskanen, Alina Solomon

**Affiliations:** 1 Institute of Clinical Medicine, Neurology, University of Eastern Finland, P.O. Box 1627, 70211 Kuopio, Finland; 2 Institute for Neuroscience, Newcastle University, Newcastle upon Tyne, UK; 3 VTT Technical Research Centre of Finland Ltd., Tampere, Finland; 4 Division of Clinical Geriatrics, NVS, Karolinska Institutet, Stockholm, Sweden; 5 Chronic Disease Prevention Unit, National Institute for Health and Welfare, Helsinki, Finland; 6 Institute of Public Health and Clinical Nutrition, University of Eastern Finland, Kuopio, Finland; 7 Combinostics, Tampere, Finland; 8 Department of Pathology, University of Helsinki and HUSLAB, Helsinki, Finland; 9 Department of Neurosurgery, University of Helsinki and Helsinki University Hospital, Helsinki, Finland

**Keywords:** Dementia, Neuropathology, Oldest old, Prediction, Supervised machine learning

## Abstract

**Background:**

We developed multifactorial models for predicting incident dementia and brain pathology in the oldest old using the Vantaa 85+ cohort.

**Methods:**

We included participants without dementia at baseline and at least 2 years of follow-up (*N* = 245) for dementia prediction or with autopsy data (*N* = 163) for pathology. A supervised machine learning method was used for model development, considering sociodemographic, cognitive, clinical, vascular, and lifestyle factors, as well as *APOE* genotype. Neuropathological assessments included β-amyloid, neurofibrillary tangles and neuritic plaques, cerebral amyloid angiopathy (CAA), macro- and microscopic infarcts, α-synuclein pathology, hippocampal sclerosis, and TDP-43.

**Results:**

Prediction model performance was evaluated using AUC for 10 × 10-fold cross-validation. Overall AUCs were 0.73 for dementia, 0.64–0.68 for Alzheimer’s disease (AD)- or amyloid-related pathologies, 0.72 for macroinfarcts, and 0.61 for microinfarcts. Predictors for dementia were different from those in previous reports of younger populations; for example, age, sex, and vascular and lifestyle factors were not predictive. Predictors for dementia versus pathology were also different, because cognition and education predicted dementia but not AD- or amyloid-related pathologies. *APOE* genotype was most consistently present across all models. *APOE* alleles had a different impact: ε4 did not predict dementia, but it did predict all AD- or amyloid-related pathologies; ε2 predicted dementia, but it was protective against amyloid and neuropathological AD; and ε3ε3 was protective against dementia, neurofibrillary tangles, and CAA. Very few other factors were predictive of pathology.

**Conclusions:**

Differences between predictors for dementia in younger old versus oldest old populations, as well as for dementia versus pathology, should be considered more carefully in future studies.

**Electronic supplementary material:**

The online version of this article (10.1186/s13195-018-0450-3) contains supplementary material, which is available to authorized users.

## Background

The oldest old constitute the largest and fastest growing population with dementia [1], but they are less often the focus of dementia prevention studies. Cohort studies with participants aged 85+ years [2–7] have investigated individual risk factors in association with dementia, but the predictive value of more complex multifactorial risk profiles in the oldest old is still unclear. Several dementia risk scores have been developed in younger populations, but they tend to perform poorly for predicting dementia in the oldest old age groups [8, 9]. The association of vascular and lifestyle-related factors with dementia risk, for example, has been shown to vary with age [10], and risk profiles predictive of subsequent dementia can differ between midlife and older age [9].

While most multifactorial prediction models or risk scores have focused on dementia, less is known about longitudinal prediction of neuropathology in people without dementia. In the oldest old, multiple dementia-related pathologies are common [11], but the association with a dementia diagnosis may be less straightforward than in younger age groups [10]. In this context, it becomes particularly important to investigate potential differences between predictors for dementia and for specific types of neuropathologies.

The main aims of the present study based on the Vantaa 85+ cohort are to develop multifactorial models for (1) predicting incident dementia in the oldest old, considering sociodemographic, cognitive, clinical, lifestyle, and apolipoprotein E (*APOE*) genotype data; and (2) predicting dementia-related neuropathologies at death in the oldest old, including Alzheimer’s disease (AD)-related pathology (amyloid plaques and neurofibrillary tangles), cerebral amyloid angiopathy (CAA), cerebral macro- and microinfarcts, and Lewy body pathology (α-synuclein).

## Methods

### Study population

The Vantaa 85+ study has been described in detail previously [4, 12]. In brief, the study focused on residents in the City of Vantaa in southern Finland who were at least 85 years old in 1991. Of the 601 people invited to participate, 11 refused, 1 could not be reached, and 1 died, leaving 588 (98%) participants who gave informed consent to participate in the study. Additionally, 35 people died before the baseline clinical examination, which was done for 553 participants. At baseline, 214 participants were diagnosed with dementia, and 339 did not have dementia. Clinical reexaminations were conducted in 1994, 1996, 1999, and 2001. At the time of death, additionally 101 participants had been diagnosed with dementia. Postmortem examination was conducted for 288 participants who attended the baseline clinical examination and 16 who had died before baseline. The Vantaa 85+ study was approved by the ethics committee of the Health Centre of the City of Vantaa. Written consent for the autopsies was given by the nearest relatives of the deceased.

**Fig. 1:**
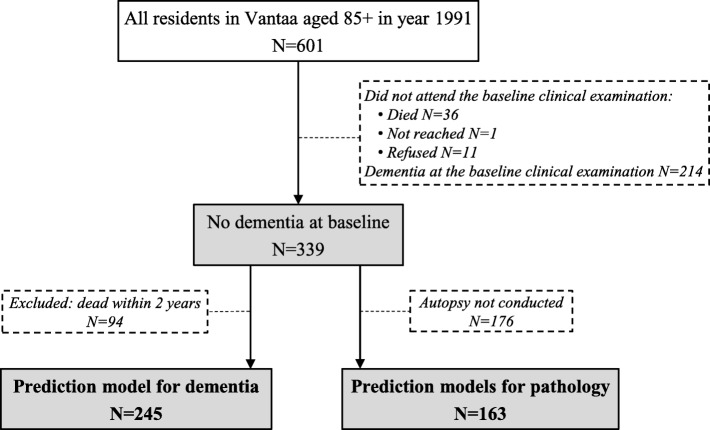
Study design flowchart

To reduce the effects of mortality, of the 339 without dementia at baseline, 94 participants who died within the first 2 years of follow-up were excluded from the dementia prediction model. This eliminated significant time-to-death differences between individuals who died with and without dementia. Of the 245 remaining participants without baseline dementia and who were included in the model development (Fig. 1), 97 subsequently developed dementia.

The study population used for neuropathological prediction model development included 163 participants who attended the baseline examination, did not have dementia at baseline, and had available autopsy data. Participants with baseline dementia were excluded to enable comparison with the dementia prediction model.

### Assessment of factors included in prediction models

Factors included in prediction models were assessed at the baseline clinical evaluation, when participants were examined by a physician and interviewed on their health, health-related behavior, and medication by a trained nurse. Medical history was additionally verified using primary health care records. Sociodemographic factors included age, sex, years of formal education, and social class [13]. Cognition was assessed with the Mini Mental State Examination (MMSE) [14] and the Short Portable Mental Status Questionnaire (SPMSQ; recorded number of errors) [15]. Participants self-reported subjective memory decline on a scale of no/a little/yes. Functional abilities were evaluated using activities of daily living (ADL) and the Instrumental Activities of Daily Living Scale (IADL) [16, 17]. Competence in daily activities was also assessed in the interview with a single question with six answer options ranging from “independent” (1 point) to “needs help in all activities” (6 points). The Zung Self-Rating Depression Scale was administered to assess depressive symptoms [18]. Comorbidities included in prediction models were diabetes, cardiovascular conditions (angina pectoris, heart infarction, atrial fibrillation, heart failure, arteriosclerosis obliterans, or hypertension) and cerebrovascular conditions (stroke or transient ischemic attack). Other vascular and lifestyle-related factors were systolic and diastolic blood pressure, body mass index (BMI), alcohol use, and smoking.

Total cholesterol as well as high-density lipoprotein (HDL) and low-density lipoprotein (LDL) cholesterol were quantified from baseline blood samples using enzymatic methods [4]. *APOE* genotyping was done with a combination of DNA minisequencing [19] and DNA amplification through PCR followed by restriction enzyme digestion with HhaI [20].

### Dementia diagnosis

Dementia was diagnosed according to the revised criteria of the *Diagnostic and Statistical Manual of Mental Disorders, Third Edition* [21]. AD and vascular dementia were diagnosed using the National Institute of Neurological and Communicative Disorders–Alzheimer’s Disease and Related Disorders Association [22] and National Institute of Neurological Disorders and Stroke–Association Internationale pour la Recherche en l’Enseignement en Neurosciences [23] criteria. Diagnosis was based on a broad range of information, including interviews, health examinations, cognitive and functional assessments, and health and social work records (e.g., information on home services or other social care services provided to participants based on diminished functional or cognitive capacity). Diagnoses were made by consensus of two neurologists.

Incident dementia cases were identified from medical and social work records, as well as from the information collected at the study follow-up visits using examinations and interviews with participants and their relatives or caregivers [24]. Although clinicians were not blinded to cognitive/functional assessments during these visits (e.g., MMSE, SPMSQ, ADL, IADL), diagnoses relied primarily on overall clinical judgment based on all available information.

### Neuropathological assessment

Neuropathological assessments have been described in detail previously [12, 24–28]. In brief, brains obtained at autopsy were fixed in phosphate-buffered 4% formaldehyde for at least 2 weeks and examined independently of clinical data. For AD-related pathology, the Consortium to Establish a Registry for Alzheimer’s disease (CERAD) protocol was followed [29]. Methenamine silver staining was used for β-amyloid [30], and the modified Bielschowsky method was used for neurofibrillary tangles and neuritic plaques [31]. As described previously, β-amyloid load was determined as the average fraction of cortical area covered by methenamine silver-stained plaques in four neocortical samples [12]. The average number of neurofibrillary tangles was also determined in the four samples [25]. The CERAD scores and Braak stages were defined as originally described [29, 32]. CAA was analyzed in six brain regions (frontal, parietal, temporal and occipital lobes, hippocampus, and cerebellum) based on Congo red staining and confirmed using IHC against β-amyloid peptide [26]. Macroscopic infarcts (cavitary lesions or solid cerebral infarcts visible to the naked eye) were identified from sliced cerebral hemispheres, brainstem, and cerebellum. All lesions were histologically ascertained to be infarcts. Cortical microinfarcts were analyzed in the H&E-stained tissue sections in the same six brain regions as CAA [26]. They were focal lesions smaller than 2 mm invisible to the naked eye with neuronal loss, glial cell and macrophage reaction, and/or cystic tissue necrosis. Sections of substantia nigra stained with the H&E method and sections of substantia nigra and hippocampus stained with antibodies against α-synuclein were used to screen for Lewy body-related pathology [27].

Hippocampal sclerosis (HS) and TDP-43 (transactive response binding protein 43) immunopositivity in the granular cell layer of the hippocampus were assessed as previously described [28]. In summary, HS and hemispheric symmetry/asymmetry were determined on H&E staining by estimating the severity of neuronal loss. The density of neurons was assessed semiquantitatively by three observers. For TDP-43 immunostaining, right-sided hippocampus tissue blocks were cut into 4-μm-thick sections and stained with the Lab Vision immunostainer (Thermo Fisher Scientific, Waltham, MA, USA), and polymer-based kits were used for detection.

### Disease State Index

The Disease State Index (DSI) is a supervised machine learning method designed for predicting disease outcomes and differential diagnostics as a clinical decision-making tool. A detailed description has been published previously [33]. Compared with traditional methods for developing dementia risk scores, DSI is able to deal with larger amounts of heterogeneous data, to handle missing data, and to use unprocessed data without prespecified cutoffs for predictors. Conceptually related factors are structured into groups, such as combining all cognitive tests. This is useful for filtering noise and preventing strongly correlated factors from being multiplied. DSI thus provides detailed information about predictive performance on multiple levels simultaneously: the independent performance of each factor, the combined performance of a group of similar factors, and the overall performance of the entire model. DSI has accuracy comparable to that of methods such as logistic regression, support vector machines, and Bayes inference [33] and has previously been used for predicting dementia [34], progression of mild cognitive impairment [33, 35, 36], and differential diagnosis of neurodegenerative diseases [37].

DSI builds a model from the distributions of data using a population with known outcomes. For tested individuals, DSI gives index values ranging from 0 to 1, describing similarity of the data to the distributions in the model. A value close to 0 indicates that the data are similar to controls (no subsequent dementia or pathology), while a value close to 1 shows similarity to cases (subsequent dementia or pathology). The data used for the predictions can be dichotomous, continuous, or categorical.

First, a fitness function is calculated for each factor. Fitness function *f*(*x*) is the share of false-negative errors divided by the sum of false-negative and false-positive errors, using measurement value *x* as a threshold for classification. It goes through the distribution using each point as a classification threshold to evaluate the shares of false-negatives and false-positives, assigning 0 to values unique to controls and 1 to values unique to cases.

To complement fitness, a relevance value is calculated for each measure. The relevance value ranges from 0 to 1 and indicates the ability, based on the data, to differentiate between cases and controls in general. Relevance is defined as the sum of sensitivity and specificity minus 1, also known as the Youden index. Two data distributions that are completely overlapping will receive a relevance of 0, while two distributions with no overlap will get a relevance of 1.

Conceptually related factors are structured into groups to combine the effect of possibly correlating factors to a single predictor. Individual factors are combined into a group DSI value through a weighted average based on their relevance values. This process is then repeated recursively for all groups to obtain a total DSI value. Any missing values are ignored as part of the model, and the total score is calculated only from the available values.

### Data analysis

We built DSI models for predicting dementia and the different neuropathologies. AUCs with 95% CIs for a 10 × 10-fold cross-validation were calculated to evaluate model performance. The dataset was divided into ten random subgroups, where nine were combined to form the training group and one acted as the test group. This process was completed for each subgroup, and the cross-validation itself was repeated ten times. Thus, we show mean AUCs and 95% CIs resulting from the 10 × 10 cross-validation process. Factor selection was conducted before the model building; that is, only factors that were significantly different (*p* < 0.05) between the groups with and without the outcome of interest were included in the final models. The initial list of factor groups and individual factors included sociodemographics (age, sex, education, social class), cognition (MMSE total score, MMSE orientation, MMSE word list - sum of registration and recall tasks, MMSE calculation, MMSE other tasks, and SPMSQ), functioning (sum of ADL and IADL, competence in daily activities question, and subjective memory decline),* APOE* genotype (binary variables: *ε2* carrier versus noncarrier, *ε4* carrier versus noncarrier, genotype *ε3ε3* versus others; and a categorical variable: all genotypes [*ε2ε2*, *ε2ε3*, *ε2ε4*, *ε3ε3*, *ε3ε4*, or *ε4ε4*]), comorbidities (cardiovascular, cerebrovascular, and diabetes), cholesterol (total, LDL, and HDL), blood pressure (systolic and diastolic), lifestyle (BMI, alcohol use, and smoking), and depressive symptoms (Zung Self-Rating Depression Scale).

The following neuropathological outcomes were dichotomized as present versus absent: β-amyloid load (average fraction of cortical area covered by methenamine silver-stained plaques > 0), tangle count (average number of neurofibrillary tangles > 0), CAA (average percentage of blood vessels with CAA > 0), cerebral macroinfarcts (total number > 0), microinfarcts (number > 0), α-synuclein (brainstem, limbic, or diffuse neocortical pathology present versus absent), HS (severe marked/total loss versus no/minor loss of pyramidal neurons in the CA1 and subiculum), and TDP-43 (immunopositivity in the granular cell layer present versus absent). Neuropathological AD was defined on the basis of National Institute on Aging–Alzheimer’s Association criteria [38] using the combination of Braak and CERAD scores, and it was dichotomized as present (intermediate or high likelihood of AD) versus absent (low likelihood of AD).

To investigate relationships between neuropathological variables and how they predict dementia, we conducted principal component analysis (PCA) in 159 participants without dementia at baseline and who had complete neuropathological data. The pca function in MATLAB R2015b (MathWorks, Natick, MA, USA) was used. The pathology variables were centered but not weighted by variance. The principal component (PC) scores of each participant were used as predictors for dementia, and predictive performance for each PC was assessed using AUC values.

## Results

### Predicting dementia

Characteristics for the dementia prediction population are shown in Table 1. The population consisted of 245 participants without dementia at baseline who were alive for at least 2 more years. Mean follow-up was 5.6 years, and 97 (40%) of the participants developed dementia before death. Education, performance in MMSE, SPMSQ, and competence in daily activities were significantly lower in people with subsequent dementia. Differences were also detected for *APOE* genotype. Other baseline characteristics were not significantly different between groups (Table 1) and were excluded from the prediction model.

**Table 1:** Baseline characteristics of the dementia prediction population

Characteristics	No dementia at death (*n* = 148)	Dementia at death (*n* = 97)	*p* Value
Follow-up time, years	5.4 (2.7)	5.8 (2.6)	0.3
Sociodemographics			
Age at baseline, years	88.4 (2.6)	88.3 (2.6)	0.7
Men, *n* (%)	33 (22%)	19 (20%)	0.6
Education, years	4.6 (3.3)	3.7 (2.0)	0.01
Social class	6.0 (1.5)	6.2 (1.3)	0.2
Cognition			
MMSE Total	25.3 (3.3)	22.2 (4.5)	< 0.001
MMSE Calculation	3.4 (1.6)	2.9 (1.6)	0.02
MMSE Orientation	9.5 (0.8)	8.7 (1.6)	< 0.001
MMSE Other tasks	7.4 (1.2)	6.7 (1.4)	< 0.001
MMSE Wordlist	5.0 (1.1)	4.2 (1.3)	< 0.001
SPMSQ	0.8 (1.4)	1.8 (1.9)	< 0.001
Functioning			
Competence in daily activities	2.6 (1.3)	3.2 (1.4)	0.001
ADL sum (ADL + IADL)	29.7 (10.2)	31.6 (10.1)	0.2
Subjective memory decline	1.7 (0.6)	1.9 (0.7)	0.05
*APOE* genotype			
*ε2ε3*	17 (12%)	18 (19%)	0.02
*ε2ε4*	1 (1%)	5 (6%)	
*ε3ε3*	101 (69%)	53 (55%)	
*ε3ε4*	27 (18%)	18 (19%)	
*ε4ε4*	0 (0%)	1 (1%)	
Comorbidity, *n* (%)			
Cardiovascular	114 (77%)	66 (68%)	0.1
Cerebrovascular	22(15%)	19 (20%)	0.3
Diabetes	29 (20%)	28 (29%)	0.09
Cholesterol, mmol/L			
Total cholesterol	5.9 (1.3)	5.7 (1.1)	0.2
LDL cholesterol	4.0 (1.2)	3.8 (1.0)	0.2
HDL cholesterol	1.0 (0.3)	1.1 (0.3)	0.2
Blood pressure, mmHg			
Systolic	161 (25)	157 (27)	0.2
Diastolic	85 (11)	84 (12)	0.6
Lifestyle factors			
BMI	25.4 (4.4)	24.9 (3.6)	0.3
No alcohol use, *n* (%)	99 (67%)	67 (69%)	0.8
Nonsmokers, *n* (%)	144 (97%)	95 (98%)	0.7
Depressive symptoms			
Zung Self-Rating Depression Scale	26.8 (5.8)	26.7 (5.5)	1

*Abbreviations*: *ADL* Activities of daily living, *APOE* Apolipoprotein E, *BMI* body mass index, *HDL/LDL* High-/low-density lipoprotein, *IADL* Instrumental activities of daily living, *MMSE* Mini Mental State Examination, *SPMSQ* Short Portable Mental Status Questionnaire

Values are shown as mean (SD) or number (percent). *p* Values were calculated with the Mann-Whitney *U* test or χ^2^ test for categorical variables. Social class is categorized on a scale from 1 (lowest) to 10 (highest) [13]

Cross-validation results for the DSI model for predicting dementia development are shown in Table 2. AUC was 0.73 for the entire model. According to AUC values for the groups of predictors, cognition including SPMSQ and both MMSE total score and its four subcategories (orientation, calculation, word list, and other tasks), were the most important predictors of dementia, followed by functioning (competence in daily activities), sociodemographics (education), and *APOE* status. *APOEε2* carrier status predicted dementia development before death, while the *ε3ε3* genotype was protective against dementia development, although AUCs were relatively low. The impact of *APOEε2* and other major predictors was similar in further analyses considering clinical diagnosis of AD and vascular dementia separately (results not shown).

**Table 2:** Dementia prediction model

Predictors	AUC [95% CI]	Sensitivity [95% CI]	Specificity [95% CI]
Entire model^a^	0.73 [0.68–0.78]	0.66 [0.63–0.69]	0.68 [0.66–0.71]
Cognition^b^	0.72 [0.66–0.78]	0.55 [0.51–0.59]	0.55 [0.52–0.58]
MMSE Calculation^c^	0.60 [0.53–0.68]	0.53 [0.49–0.56]	0.68 [0.66–0.70]
MMSE Orientation^c^	0.64 [0.58–0.70]	0.68 [0.65–0.71]	0.54 [0.51–0.56]
MMSE Other tasks^c^	0.65 [0.58–0.72]	0.56 [0.53–0.58]	0.67 [0.65–0.69]
MMSE Wordlist^c^	0.68 [0.62–0.75]	0.77 [0.75–0.80]	0.57 [0.55–0.60]
SPMSQ^c^	0.71 [0.65–0.77]	0.67 [0.65–0.70]	0.64 [0.62–0.67]
MMSE total^c^	0.71 [0.64–0.77]	0.62 [0.59–0.65]	0.61 [0.58–0.63]
Functioning^b^	0.61 [0.55–0.67]	0.62 [0.59–0.65]	0.61 [0.58–0.63]
Competence in daily activities^c^	0.61 [0.55–0.67]	0.83 [0.81–0.86]	0.35 [0.32–0.38]
Sociodemographics^b^	0.60 [0.54–0.65]	0.83 [0.81–0.86]	0.35 [0.32–0.38]
Education, years^c^	0.60 [0.54–0.65]	0.66 [0.63–0.69]	0.68 [0.66–0.71]
*APOE* genotype^b^	0.58 [0.52–0.64]	0.45 [0.42–0.47]	0.69 [0.67–0.71]
*APOEε2* carriers^c^	0.56 [0.51–0.61]	0.25 [0.23–0.27]	0.88 [0.86–0.89]
*APOEε3ε3* genotype^c^	0.57 [0.51–0.63]	0.45 [0.42–0.47]	0.69 [0.67–0.71]
All genotypes^c,d^ (*23*/*24*/*33*/*34*/*44*)	0.58 [0.51–0.64]	0.67 [0.64–0.70]	0.66 [0.63–0.68]

*Abbreviations*: *APOE* Apoliprotein E, *MMSE* Mini Mental State Examination, *SPMSQ* Short Portable Mental Status Questionnaire

AUC, sensitivity and specificity [95% CI] values using the cutoff point Disease State Index (DSI) = 0.5 are shown for 10 × 10-fold cross-validation of the DSI model. Numbers of participants with missing data were 3 for education and 3 for APOE genotype

^a^Overall model performance

^b^Overall performance of each group of related predictors

^c^Performance of each individual predictor

^d^Categorical variable including genotype *ε2ε3*, *ε2ε4*, *ε3ε3*, *ε3ε4*, or *ε4ε4*

### Predicting pathology

For predicting pathology, we included the 163 participants with no dementia at baseline and available autopsy data. This population had a mean age of 88.7 years, a follow-up time of 4.1 years, and 4.3 years of education. Thirty-one (19%) of these participants were male, 33 (21%) of them were *APOEε4* carriers, and 26 (17%) were *ε2* carriers. Fifty-nine (36%) had dementia at death.

Cross-validation results (AUCs) of the DSI pathology prediction models are shown in Table 3. Sensitivities and specificities are shown in Additional file 1: Table S1. The total AUCs for AD- or amyloid-related pathologies were 0.66 for amyloid load, 0.64 for tangle count, 0.68 for neuropathological AD, and 0.66 for CAA. *APOE* genotype had the highest AUCs for all these pathologies, but there were differences in the impact of different alleles. *APOEε4* carrier status was predictive for all four pathology outcomes, while *APOEε2* carrier status was protective against β-amyloid load and neuropathological AD. The *ε3ε3* genotype was protective against tangle count and CAA, but it was not related to amyloid load or neuropathological AD.

**Table 3:** Neuropathology prediction models

Neuropathological outcomes	Predictors	AUC [95%CI]	Description of predictors by neuropathological outcome categories	
			*Absent*	*Present*
β-Amyloid load	Overall model^a^	0.66 [0.56 - 0.77]	N=37 (23%)	N=126 (77%)
	APOE genotype^b^	0.63 [0.56 - 0.71]		
	*APOEε2* carriers^c^	0.57 [0.50 - 0.64]	10 (27%)	16 (13%)
	*APOEε4* carriers^c^	0.60 [0.55 - 0.65]	2 (5%)	31 (25%)
	All genotypes (*23/24/33/34/44*)^c,d^	0.62 [0.54 - 0.70]	9/1/26/1/0 (24/3/70/3/0%)	13/3/78/28/0 (11/2/64/23/0%)
	Functioning^b^	0.61 [0.51 - 0.70]		
	Competence in Daily Activities^c^	0.61 [0.51 - 0.70]	2.7 (1.4)	3.2 (1.4)
Tangle count	Overall model^a^	0.64 [0.55 - 0.73]	N=64 (39%)	N=99 (61%)
	APOE genotype^b^	0.60 [0.54 - 0.67]		
	*APOEε4* carriers^c^	0.61 [0.55 - 0.67]	5 (8%)	28 (29%)
	*APOE ε3ε3* genotype^c^	0.59 [0.52 - 0.66]	48 (75%)	56 (58%)
	All genotypes (*23/24/33/34/44*)^c,d^	0.55 [0.48 - 0.62]	10/1/48/4/0 (16/2/76/6/0%)	12/3/56/25/0 (13/3/58/26/0%)
	Cholesterol^b^	0.60 [0.51 - 0.70]		
	Total^c^	0.61 [0.51 - 0.70]	5.6 (1.4)	5.9 (1.2)
	LDL^c^	0.60 [0.50 - 0.69]	3.6 (1.1)	3.9 (1.0)
	Functioning^b^	0.59 [0.50 - 0.67]		
	Subjective memory decline^c^	0.59 [0.50 - 0.67]	1.7 (0.6)	1.9 (0.7)
Neuropathological AD	Overall model^a^	0.68 [0.61 - 0.76]	N=86 (53%)	N=77 (47%)
	APOE genotype^b^	0.65 [0.59 - 0.71]		
	*APOEε2* carriers^c^	0.57 [0.51 - 0.62]	19 (23%)	7 (9%)
	*APOEε4* carriers^c^	0.62 [0.57 - 0.67]	8 (10%)	25 (33%)
	All genotypes (*23/24/33/34/44*)^c,d^	0.64 [0.58 - 0.70]	17/2/59/6/0 (20/2/70/7/0%)	5/2/45/23/0 (7/3/60/31/0%)
	Sociodemographics^b^	0.62 [0.53 - 0.70]		
	Social class^c^	0.62 [0.53 - 0.70]	6.4 (1.5)	5.9 (1.2)
	Functioning^b^	0.58 [0.50 - 0.66]		
	Subjective memory decline^c^	0.58 [0.50 - 0.66]	1.7 (0.6)	1.9 (0.7)
Cerebral amyloid angiopathy	Overall model^a^	0.66 [0.58 - 0.74]	N=56 (35%)	N=103 (65%)
	APOE genotype^b^	0.62 [0.55 - 0.69]		
	*APOEε4* carriers^c^	0.63 [0.58 - 0.68]	2 (4%)	30 (30%)
	*APOE ε3ε3* genotype^c^	0.59 [0.52 - 0.67]	43 (78%)	59 (59%)
	All genotypes (*23/24/33/34/44*)^c,d^	0.61 [0.55 - 0.68]	10/1/43/1/0 (18/2/78/2/0%)	11/3/59/27/0 (11/3/59/27/0%)
	Comorbidity^b^	0.59 [0.52 - 0.65]		
	Cardiovascular^c^	0.59 [0.52 - 0.65]	48 (86%)	70 (68%)
	Sociodemographics	0.58 [0.53 - 0.64]		
	Gender, men^c^	0.58 [0.53 - 0.64]	5 (9%)	26 (25%)
Cerebral macroinfarcts	Overall model^a^	0.72 [0.64 - 0.79]	N=83 (51%)	N=80 (49%)
	Comorbidity^b^	0.64 [0.58 - 0.70]		
	Cerebrovascular^c^	0.64 [0.58 - 0.70]	4 (5%)	26 (33%)
	Cognition^b^	0.63 [0.54 - 0.72]		
	MMSE Wordlist^c^	0.63 [0.55 - 0.71]	4.6 (1.3)	4.0 (1.3)
	MMSE Total^c^	0.62 [0.52 - 0.71]	23.8 (4.6)	22.0 (4.5)
	Lifestyle^b^	0.62 [0.53 - 0.70]		
	BMI^c^	0.62 [0.53 - 0.70]	23.9 (4.1)	25.5 (4.2)
	Functioning^b^	0.59 [0.51 - 0.66]		
	Competence in Daily Activities^c^	0.59 [0.51 - 0.66]	2.9 (1.4)	3.3 (1.3)
Cortical macroinfarcts	Overall model^a^	0.71 [0.63 - 0.79]	N=116 (71%)	N=47 (29%)
	Comorbidity^b^	0.64 [0.57 - 0.71]		
	Cerebrovascular^c^	0.64 [0.57 - 0.71]	12 (10%)	18 (38%)
	APOE genotype^b^	0.60 [0.51 - 0.69]		
	*APOEε4* carriers^c^	0.58 [0.51 - 0.65]	18 (16%)	15 (33%)
	*APOE ε3ε3* genotype^c^	0.59 [0.50 - 0.68]	80 (71%)	24 (52%)
White matter macroinfarcts	Overall model^a^	0.76 [0.65 - 0.87]	N=140 (86%)	N=23 (14%)
	Cholesterol^b^	0.72 [0.60 - 0.83]		
	HDL^c^	0.68 [0.58 - 0.79]	1.0 (0.3)	0.9 (0.3)
	LDL^c^	0.70 [0.58 - 0.83]	3.9 (1.0)	3.2 (1.0)
	Comorbidity^b^	0.62 [0.51 - 0.73]		
	Cerebrovascular^c^	0.62 [0.51 - 0.73]	21 (15%)	9 (39%)
	APOE genotype^b^	0.61 [0.50 - 0.71]		
	*APOEε2* carriers^c^	0.61 [0.50 - 0.71]	18 (13%)	8 (35%)
Cerebral microinfarcts	Overall model^a^	0.61 [0.51 - 0.71]	N=130 (83%)	N=26 (17%)
	Education years^c^	0.61 [0.51 - 0.71]	4.5 (2.9)	3.3 (1.8)
Hippocampal Sclerosis	Overall model^a^	0.78 [0.64 - 0.91]	N=151 (93%)	N=11 (7%)
	Cognition^b^	0.75 [0.59 - 0.92]		
	MMSE Wordlist^c^	0.68 [0.54 - 0.83]	4.4 (1.3)	3.6 (0.9)
	MMSE Other tasks^c^	0.74 [0.57 - 0.90]	6.9 (1.5)	5.8 (1.4)
	MMSE Total^c^	0.72 [0.55 - 0.90]	23.1 (4.6)	22.3 (4.1)
	Lifestyle^b^	0.57 [0.42 - 0.71]		
	Current smoking^c^	0.57 [0.42 - 0.71]	4 (3%)	2 (18%)
TDP-43	Overall model^a^	0.69 [0.56 - 0.81]	N=139 (86%)	N=21 (13%)
	Zung depression scale^c^	0.69 [0.56 - 0.81]	27.5 (5.8)	23.7 (2.8)

*Abbreviations*: AD Alzheimer’s Disease, *APOE* Apolipoprotein E, BMI body mass index, HDL/LDL High/low density lipoprotein, MMSE Mini-Mental State Examination, SPMSQ Short portable mental status questionnaire, TDP-43 TAR DNA-binding protein 43.

AUC [95% CI] values are shown for 10*10-fold cross-validation of the DSI model. In the “Description of predictors…” columns, values are shown as mean (standard deviation) or number (percentage). Number of participants with missing data was 4 for cerebral amyloid angiopathy, 4 for cerebral microinfarcts, , 4 for APOE genotype, 5 for MMSE, 6 for subjective memory, 3 for education, 1 for social class, 1 for smoking, 3 for Zung scale, 10 for cholesterol, and 39 for BMI.

^a^Overall model performance for each neuropathological outcome

^b^Overall performance of each group of related predictors.

^c^Performance of each individual predictor

^d^Categorical variable including genotype *ε2ε3, ε2ε4, ε3ε3, ε3ε4 or ε4ε4*

Very few other factors had predictive value (Table 3): poorer competence in daily activities for β-amyloid load, higher total and LDL cholesterol, and subjective memory decline for tangle count; lower social class and subjective memory decline for neuropathological AD; and absence of cardiovascular comorbidity and male sex for CAA.

The model for cerebral macroinfarcts had the best predictive performance with total AUC of 0.72 (Table 3). The predictors in descending order of AUC values were history of cerebrovascular conditions, poorer MMSE score (total and word list learning and recall tasks), higher BMI, and poorer competence in daily activities. We also modeled the two most common subtypes of cerebral macroinfarcts: cortical and white matter. Cortical macroinfarcts (AUC of 0.71) were predicted by cerebrovascular comorbidity and *APOE* genotype. *APOEε4* carriers were more likely to develop cortical macroinfarcts, while genotype ε3ε3 was protective. White matter macroinfarcts (AUC of 0.76) were predicted by cholesterol, cerebrovascular comorbidity, and *APOEε2* carrier status. The AUC for the cerebral microinfarcts model was 0.61, with education as the only predictor.

HS (AUC of 0.78) was predicted by cognition (MMSE total score, word list learning and recall, and other tasks). Current smokers were also more likely to have HS (Table 3). TDP-43 was only predicted by less pronounced depressive symptoms (AUC of 0.69). There were no significant predictors found for α-synuclein.

**Fig. 2:**
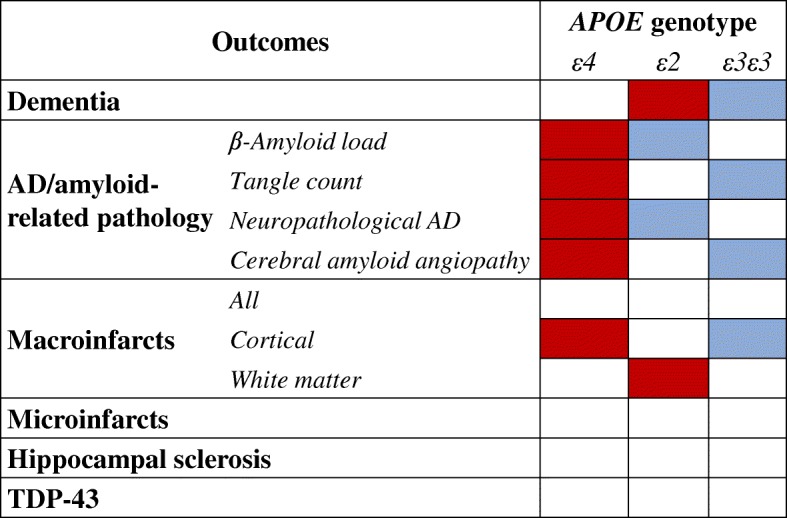
Impact of apolipoprotein E (*APOE*) genotype on dementia versus neuropathology. *Red* indicates alleles that predicted dementia/pathology. *Blue* indicates alleles that were protective against dementia/pathology. *White* indicates alleles with no significant impact

Overall, *APOE* genotype was the predictor that emerged most consistently across all models. The impact of *APOE* on dementia versus pathology is summarized in Fig. 2.

Associations between pathology and dementia at death in participants without dementia at baseline are shown in Additional file 1: Table S2, and results of the PCA analysis are provided in Additional file 1: Table S3. The first three components of PCA explained 56% of the variance in autopsy findings and reflected three mostly independent pathological processes: AD-/amyloid-related pathology, including β-amyloid, neurofibrillary tangles, and CAA (PC1); vascular-type pathology, including primarily macroscopic infarcts (PC2); and Lewy body-type pathology, including α-synuclein (PC3). PC1 was most predictive of dementia (AUC of 0.71), followed by PC2 (AUC of 0.60). The other PCs did not predict dementia.

## Discussion

### Predicting dementia in the oldest old

The DSI model performance for predicting dementia onset before death, on average 6 years later, in people aged 85+ years was close to the 10-year DSI dementia prediction model in a younger-old population [34] and was in the upper range of reported performance for previous dementia risk scores in younger populations [9]. Similarly to other dementia risk scores [9, 34], cognition was the main predictor, followed by functioning and education levels. However, there were several important differences compared with younger populations. Age, sex, and vascular and lifestyle factors were not predictive of dementia in the present study, although they are usually important predictors in midlife. The age range for 85+ populations is inherently smaller than for younger cohorts, potentially limiting the predictive value of age. Individuals who survive to the age of 85 years without dementia are also a selected group. While mechanisms are not fully clear, associations of vascular and lifestyle factors with dementia have been reported to differ in midlife versus late life [10].

*APOE* genotype was related to incident dementia, but the pattern was different from that in younger populations, where the *ε4* allele increases dementia risk, while the *ε2* allele seems protective (www.alzgene.org). In the present study, *APOEε4* carrier status was not important for dementia prediction, in line with previous findings in the oldest old [39, 40]. The *ε3ε3* genotype was protective, while the *ε2* allele was predictive of subsequent dementia. Compared with younger populations, a lower proportion of *ε4* carriers and a higher proportion of *ε2* carriers have been reported in the oldest old [10, 40], including the Vantaa 85+ cohort [41]. Three previous population-based studies with shorter follow-up than Vantaa 85+ reported no protective effect of the *ε2* allele against incident dementia after the age of 85 years [40, 42, 43]. Additionally, the *ε2* allele increased the risk of incident vascular dementia in one study [42]. Previous reports on lower risk of dementia among the oldest old *APOEε2 *carriers have come from cross-sectional studies of dementia prevalence at death [44], and this may not necessarily apply longitudinally to dementia incidence after the age of 85 years.

### Predicting dementia versus predicting neuropathology

The *APOE* genotype consistently predicted AD- or amyloid-related pathologies at death on average 6 years later, but with a different pattern than for incident dementia. The *ε4* allele predicted all these pathologies. *ε3ε3* genotype was protective against neurofibrillary tangles and CAA. The *ε2* allele was protective against β-amyloid load and neuropathological AD. This pattern is closer to findings derived from younger-old populations, where the *ε4* allele increases the risk and *ε2* allele decreases the risk of subsequent AD-related pathology [45]. A conflicting finding was reported in the 90+ Study, where *ε2* carriers had increased CERAD scores in cross-sectional analyses at death [44]. However, further analyses showed lower cortical β-amyloid percentage areas in *ε2* carriers [46].

While APOE was not related to cerebral macroinfarcts in general in the present study, the *ε4* allele predicted cortical macroinfarcts, and the *ε2* allele predicted white matter macroinfarcts. A meta-analysis of studies in younger populations has linked both the *ε4* and *ε2* alleles to increasing burden in magnetic resonance imaging markers of cerebrovascular disease, including white matter hyperintensities [47]. However, longitudinal associations of *APOE* genotype with subsequent cerebrovascular lesions in the oldest old are still unclear. In the Vantaa 85+ study, while white matter infarcts alone were not significantly related to dementia diagnosis at death, they may suggest a potential explanation for the predictive effect of *APOEε2* on incident dementia.

Very few other factors besides *APOE* predicted neuropathology. Vascular and lifestyle factors did not predict β-amyloid load or neuropathological AD. It is still debated whether vascular and lifestyle risk factors for dementia are actually related to amyloid pathology and whether such relationships may be age-dependent. Our finding that higher LDL and total cholesterol predicted tangle count needs to be verified in other 85+ cohorts.

Cognitive performance was not predictive of AD- or amyloid-related pathologies, although it predicted dementia, cerebral macroinfarcts, and HS. Of the included sociodemographic factors, only lower social class predicted neuropathological AD.

Predictors for HS and TDP-43 pathology are still unclear. While current smoking was related to HS and less pronounced depressive symptoms were related to TDP-43, the number of participants with these pathologies was very small in this study, and these findings require verification in other cohorts.

Overall, predictive performance of the models (AUC, sensitivity, specificity) was not very high. While study-specific limitations may have contributed to this, it is also possible that neither incident dementia nor specific neuropathologies can be predicted with very high accuracy in the oldest old using predictors commonly emphasized in younger-old populations. This is also suggested by the failure of previous dementia risk scores when extrapolated from younger-old to oldest-old populations. Different approaches may be needed that better account for the heterogeneity and multipathology often existing within the 85+ age group.

### Strengths and limitations of the present study

The main strength of the present study is the prospective population-based design with a high autopsy rate over 10 years, the inclusion of participants aged > 85 years, and the multicomponent longitudinal prediction models for both dementia and specific neuropathologies. However, the developed prediction models are applicable only to a highly selected group of individuals who survive to the age of 85 years without developing dementia. External validation in other oldest-old cohorts will also be needed. The Vantaa 85+ population may differ from populations that are currently 85+ years old (e.g., for relatively low education). Health-related measures prior to the age of 85 years were not available. Sample size may have limited statistical power, especially for pathological outcomes with smaller numbers of participants.

Participants with autopsy were more likely to have incident dementia and lower MMSE at baseline than those without autopsy, which may have affected the pathology models. Participants with dementia at baseline were excluded from pathology models, but owing to their old age, some pathology may have been present at baseline. Quantitative, systematic methods were used for neuropathological assessments, but findings based on traditional silver-staining methods may be somewhat different from IHC methods for AD pathology. Other pathologies, such as aging-related tau astrogliopathy, could not be included owing to lack of data.

## Conclusions

This is the first study combining longer-term dementia and neuropathology multicomponent prediction models among the oldest old. The dementia risk profile in this age group was very different from risk profiles previously described at younger ages. Predictors of dementia did not necessarily predict pathology. *APOE* genotype was the most consistent predictor across all models, but with different impact for different alleles.

The predictive models in the present study were developed for early identification of individuals with elevated risk of subsequent dementia. Longitudinal prediction models in the oldest old are more complex than in younger-old populations, and multifactorial risk profiles including both genetic and nongenetic factors need to be further investigated.

## Additional file

Additional file 1: **Table S1.** Sensitivity and specificity of neuropathology prediction models. **Table S2.** Neuropathology characteristics by dementia status at death for participants without dementia at baseline. **Table S3.** The first three components of PCA for autopsy findings and prediction of dementia at death for participants with complete autopsy data and no dementia at baseline. (PDF 40 kb)

## Abbreviations

AD: Alzheimer’s disease
ADL: Activities of daily living
*APOE*: Apolipoprotein E
BMI: Body mass index
CAA: Cerebral amyloid angiopathy
CERAD: Consortium to Establish a Registry for Alzheimer’s Disease
DSI: Disease State Index
HDL: High-density lipoprotein
HS: Hippocampal sclerosis
IADL: Instrumental Activities of Daily Living Scale
LDL: Low-density lipoprotein
MMSE: Mini Mental State Examination
PCA: Principal component analysis
SPMSQ: Short Portable Mental Status Questionnaire
TDP-43: Transactive response binding protein 43
