# Supplementary material for: Prediction models for dementia and neuropathology in the oldest old: the Vantaa 85+ cohort study
Source: Alzheimers Res Ther. 2019 Jan 22;11:11. doi: 10.1186/s13195-018-0450-3 (PMC6343349; doi:10.1186/s13195-018-0450-3)
Supplement: Supplementary file 1 — Table S1. Sensitivity and specificity of neuropathology prediction models. Table S2. Neuropathology characteristics by dementia status at death for participants without dementia at baseline. Table S3. The first three components of PCA for autopsy findings and prediction of dementia at death for participants with complete autopsy data and no dementia at baseline. (PDF 40 kb) [file 13195_2018_450_MOESM1_ESM.pdf]

Table S1 Sensitivity and specificity for neuropathology prediction models.

Neuropathological outcomes	Predictors	Sensitivity [95%CI]	Specificity [95%CI]
β-Amyloid load	Overall model[a]	0.62 [0.52 - 0.72]	0.63 [0.44 - 0.82]
	APOE genotype[b]	0.28 [0.14 - 0.43]	0.86 [0.67 - 1.04]
	APOEε2 carriers[c]	0.87 [0.80 - 0.94]	0.27 [0.07 - 0.47]
	APOEε4 carriers[c]	0.25 [0.16 - 0.35]	0.95 [0.86 - 1.03]
	All genotypes[c,d]	0.24 [0.14 - 0.33]	0.94 [0.83 - 1.04]
	Functioning[b]	0.60 [0.50 - 0.70]	0.55 [0.36 - 0.73]
	Competence in Daily Activities[c]	0.60 [0.50 - 0.70]	0.55 [0.36 - 0.73]
Tangle count	Overall model[a]	0.58 [0.45 - 0.70]	0.56 [0.42 - 0.70]
	APOE genotype[b]	0.41 [0.29 - 0.52]	0.76 [0.65 - 0.88]
	APOEε4 carriers[c]	0.29 [0.18 - 0.40]	0.92 [0.85 - 0.99]
	APOE ε3ε3 genotype[c]	0.42 [0.30 - 0.53]	0.76 [0.65 - 0.87]
	All genotypes[c,d]	0.28 [0.18 - 0.39]	0.91 [0.82 - 1.00]
	Cholesterol[b]	0.59 [0.48 - 0.71]	0.57 [0.41 - 0.72]
	Total[c]	0.60 [0.48 - 0.71]	0.61 [0.45 - 0.76]
	LDL[c]	0.56 [0.44 - 0.69]	0.57 [0.42 - 0.72]
	Functioning[b]	0.71 [0.61 - 0.82]	0.40 [0.27 - 0.53]
	Subjective memory decline[c]	0.71 [0.61 - 0.82]	0.40 [0.27 - 0.53]
Neuropathological AD	Overall model[a]	0.57 [0.44 - 0.70]	0.63 [0.51 - 0.76]
	APOE genotype[b]	0.32 [0.20 - 0.45]	0.90 [0.83 - 0.98]
	APOEε2 carriers[c]	0.91 [0.83 - 0.98]	0.22 [0.12 - 0.33]
	APOEε4 carriers[c]	0.33 [0.21 - 0.46]	0.90 [0.83 - 0.98]
	All genotypes[c,d]	0.32 [0.19 - 0.44]	0.90 [0.83 - 0.98]
	Sociodemographics[b]	0.63 [0.49 - 0.78]	0.51 [0.40 - 0.63]
	Social class[c]	0.63 [0.49 - 0.78]	0.51 [0.40 - 0.63]
	Functioning[b]	0.73 [0.62 - 0.85]	0.39 [0.25 - 0.52]
	Subjective memory decline[c]	0.73 [0.62 - 0.85]	0.39 [0.25 - 0.52]
Cerebral amyloid angiopathy	Overall model[a]	0.66 [0.55 - 0.77]	0.62 [0.48 - 0.77]
	APOE genotype[b]	0.35 [0.24 - 0.45]	0.81 [0.69 - 0.94]
	APOEε4 carriers[c]	0.30 [0.19 - 0.41]	0.96 [0.91 - 1.02]
	APOE ε3ε3 genotype[c]	0.41 [0.31 - 0.51]	0.78 [0.67 - 0.90]
	All genotypes[c,d]	0.30 [0.19 - 0.40]	0.96 [0.91 - 1.02]
	Comorbidity[b]	0.32 [0.20 - 0.44]	0.86 [0.74 - 0.97]
	Cardiovascular[c]	0.32 [0.20 - 0.44]	0.86 [0.74 - 0.97]
	Sociodemographics	0.25 [0.14 - 0.36]	0.91 [0.82 - 1.00]
	Gender, men[c]	0.25 [0.14 - 0.36]	0.91 [0.82 - 1.00]
Cerebral macroinfarcts	Overall model[a]	0.59 [0.45 - 0.72]	0.65 [0.53 - 0.77]
	Comorbidity[b]	0.33 [0.21 - 0.44]	0.95 [0.90 - 1.00]
	Cerebrovascular[c]	0.33 [0.21 - 0.44]	0.95 [0.90 - 1.00]
	Cognition[b]	0.61 [0.45 - 0.77]	0.58 [0.44 - 0.71]
	MMSE Wordlist[c]	0.63 [0.50 - 0.76]	0.60 [0.47 - 0.72]
	MMSE Total[c]	0.60 [0.47 - 0.74]	0.63 [0.51 - 0.75]
	Lifestyle[b]	0.55 [0.37 - 0.72]	0.60 [0.44 - 0.75]
	BMI[c]	0.55 [0.37 - 0.72]	0.60 [0.44 - 0.75]
	Functioning[b]	0.65 [0.54 - 0.76]	0.52 [0.41 - 0.63]
	Competence in Daily Activities[c]	0.65 [0.54 - 0.76]	0.52 [0.41 - 0.63]
Cortical macroinfarcts	Overall model[a]	0.62 [0.44 - 0.80]	0.67 [0.56 - 0.78]
	Comorbidity[b]	0.38 [0.22 - 0.55]	0.90 [0.83 - 0.96]
	Cerebrovascular[c]	0.38 [0.22 - 0.55]	0.90 [0.83 - 0.96]
	APOE genotype[b]	0.48 [0.32 - 0.64]	0.71 [0.62 - 0.79]
	APOEε4 carriers[c]	0.33 [0.18 - 0.48]	0.84 [0.76 - 0.92]
	APOE ε3ε3 genotype[c]	0.48 [0.32 - 0.64]	0.71 [0.62 - 0.79]

White matter macroinfarcts	Overall model[a]	0.61 [0.37 - 0.86]	0.67 [0.59 - 0.75]
	Cholesterol[b]	0.68 [0.45 - 0.91]	0.65 [0.56 - 0.74]
	HDL[c]	0.62 [0.36 - 0.89]	0.60 [0.51 - 0.68]
	LDL[c]	0.63 [0.35 - 0.92]	0.60 [0.50 - 0.71]
	Comorbidity[b]	0.39 [0.19 - 0.60]	0.85 [0.79 - 0.91]
	Cerebrovascular[c]	0.39 [0.19 - 0.60]	0.85 [0.79 - 0.91]
	APOE genotype[b]	0.34 [0.15 - 0.54]	0.87 [0.81 - 0.93]
	APOEε2 carriers[c]	0.34 [0.15 - 0.54]	0.87 [0.81 - 0.93]
Cerebral microinfarcts	Overall model[a]	0.38 [0.11 - 0.66]	0.68 [0.50 - 0.86]
	Education years[c]	0.38 [0.11 - 0.66]	0.68 [0.50 - 0.86]
Hippocampal Sclerosis	Overall model[a]	0.73 [0.40 – 1.00]	0.69 [0.60 - 0.77]
	Cognition[b]	0.73 [0.41 – 1.00]	0.67 [0.58 - 0.76]
	MMSE Wordlist[c]	0.73 [0.40 - 1.00]	0.64 [0.55 - 0.73]
	MMSE Other tasks[c]	0.82 [0.53 - 1.00]	0.65 [0.56 – 0.75]
	MMSE Total[c]	0.73 [0.41 - 1.00]	0.63 [0.53 – 0.73]
	Lifestyle[b]	0.17 [0.00 - 0.43]	0.96 [0.92 – 1.00]
	Current smoking[c]	0.17 [0.00 - 0.43]	0.96 [0.92 – 1.00]
TDP-43	Overall model[a]	0.68 [0.46 - 0.89]	0.63 [0.53 - 0.72]
	Zung depression scale[c]	0.68 [0.46 - 0.89]	0.63 [0.53 - 0.72]

Abbreviations: AD Alzheimer's Disease, *APOE* Apolipoprotein E, BMI body mass index, HDL/LDL High/low density lipoprotein, MMSE Mini-Mental State Examination, SPMSQ Short portable mental status questionnaire, TDP-43 TAR DNA-binding protein 43.

Sensitivity and specificity [95% CI] values using the cut-off point DSI=0.5 are shown for 10*10-fold cross-validation of the DSI model. Number of participants with missing data was 4 for cerebral amyloid angiopathy, 4 for cerebral microinfarcts, , 4 for APOE genotype, 5 for MMSE, 6 for subjective memory, 3 for education, 1 for social class, 1 for smoking, 3 for Zung scale, 10 for cholesterol, and 39 for BMI.

[a]Overall model performance for each neuropathological outcome
[b]Overall performance of each group of related predictors.
[c]Performance of each individual predictor
[d]Categorical variable including genotype *ε2ε3, ε2ε4, ε3ε3, ε3ε4 or ε4ε4*

Table S2 Neuropathology characteristics by dementia status at death for participants without dementia at baseline.

	No dementia (N=104)	Dementia (N=59)	*p* value
β-amyloid load	74 (71%)	52 (88%)	0.02
Tangle count	55 (53%)	44 (75%)	0.008
Neuropathological AD	39 (38%)	38 (64%)	0.001
Cerebral amyloid angiopathy	59 (58%)	44 (76%)	0.04
Cerebral macroinfarcts	47 (45%)	33 (56%)	0.2
Cortical macroinfarcts	23 (22%)	24 (41%)	0.02
White matter macroinfarcts	14 (14%)	9 (15%)	0.8
Cerebral microinfarcts	16 (16%)	11 (19%)	0.7
α-synuclein	26 (25%)	22 (37%)	0.1
Hippocampal sclerosis	2 (2%)	9 (15%)	0.002
TDP-43	8 (8%)	14 (24%)	0.007

Abbreviations: AD Alzheimer's Disease, TDP-43 TAR DNA-binding protein 43.

Values are shown as number (percentage). *P* values are calculated with Fisher's exact test. Number of participants with missing data was 4 for cerebral amyloid angiopathy and for cerebral microinfarcts.

Table S3 The first three components of principal component analysis for autopsy findings and prediction of dementia at death for participants with complete autopsy data and no dementia at baseline.

All subjects	PC1	PC2	PC3
Explained variance	25%	20%	11%
AUC of PC	0.71	0.60	0.54
β-amyloid load	0.41	0.03	-0.31
Tangle count	0.48	0.00	0.38
Neuropathological AD	0.59	-0.07	-0.04
Cerebral amyloid angiopathy	0.47	-0.03	-0.18
Cerebral macroinfarcts	-0.01	0.73	-0.13
Cortical macroinfarcts	0.07	0.59	-0.15
White matter macroinfarcts	-0.08	0.24	0.01
Cerebral microinfarcts	0.11	0.10	0.16
α-synuclein	0.05	0.20	0.81
Hippocampal sclerosis	0.00	0.04	-0.02
TDP-43	0.06	0.01	0.02

Abbreviations: AD Alzheimer's Disease, PC Principal component, TDP-43 TAR DNA-binding protein 43.
